# Bedaquiline resistance probability to guide treatment decision making for rifampicin-resistant tuberculosis: insights from a qualitative study

**DOI:** 10.1186/s12879-022-07865-7

**Published:** 2022-11-22

**Authors:** Pham Hien Trang Tu, Degefaye Zelalem Anlay, Anzaan Dippenaar, Emilyn Costa Conceição, Jasna Loos, Annelies Van Rie

**Affiliations:** 1 Department of Family Medicine and Population Health, Faculty of Medicine and Health Sciences, University of Antwerp, Doornstraat 331, 2610 Antwerp, Belgium; 2 Department of Community Health Nursing, School of Nursing, College of Medicine and Health Science, University of Gondar, Gondar, Ethiopia; 3 Department of Science and Innovation, National Research Foundation Centre of Excellence for Biomedical Tuberculosis Research, South African Medical Research Council Centre for Tuberculosis Research, Division of Molecular Biology and Human Genetics, Faculty of Medicine and Health Sciences, Stellenbosch University, Cape Town, South Africa; 4 Dean’s Office, Faculty of Medicine and Health Sciences, University of Antwerp, Antwerp, Belgium

**Keywords:** Rifampicin-resistant tuberculosis, Bedaquline, Resistance probability, Treatment decision

## Abstract

**Background:**

Bedaquiline (BDQ) is a core drug for rifampicin-resistant tuberculosis (RR-TB) treatment. Accurate prediction of a BDQ-resistant phenotype from genomic data is not yet possible. A Bayesian method to predict BDQ resistance probability from next-generation sequencing data has been proposed as an alternative.

**Methods:**

We performed a qualitative study to investigate the decision-making of physicians when facing different levels of BDQ resistance probability. Fourteen semi-structured interviews were conducted with physicians experienced in treating RR-TB, sampled purposefully from eight countries with varying income levels and burden of RR-TB. Five simulated patient scenarios were used as a trigger for discussion. Factors influencing the decision of physicians to prescribe BDQ at macro-, meso- and micro levels were explored using thematic analysis.

**Results:**

The perception and interpretation of BDQ resistance probability values varied widely between physicians. The limited availability of other RR-TB drugs and the high cost of BDQ hindered physicians from altering the BDQ-containing regimen and incorporating BDQ resistance probability in their decision-making. The little experience with BDQ susceptibility testing and whole-genome sequencing results, and the discordance between phenotypic susceptibility and resistance probability were other barriers for physicians to interpret the resistance probability estimates. Especially for BDQ resistance probabilities between 25% and 70%, physicians interpreted the resistance probability value dynamically, and other factors such as clinical and bacteriological treatment response, history of exposure to BDQ, and resistance profile were often considered more important than the BDQ probability value for the decision to continue or stop BDQ. In this grey zone, some physicians opted to continue BDQ but added other drugs to strengthen the regimen.

**Conclusions:**

This study highlights the complexity of physicians' decision-making regarding the use of BDQ in RR-TB regimens for different levels of BDQ resistance probability.. Ensuring sufficient access to BDQ and companion drugs, improving knowledge of the genotype–phenotype association for BDQ resistance, availability of a rapid molecular test, building next-generation sequencing capacity, and developing a clinical decision support system incorporating BDQ resistance probability will all be essential to facilitate the implementation of BDQ resistance probability in personalizing treatment for patients with RR-TB.

**Supplementary Information:**

The online version contains supplementary material available at 10.1186/s12879-022-07865-7.

## Introduction

In 2012, Bedaquiline (BDQ) was the first new tuberculosis drug approved by the United States Food and Drug Administration in nearly 50 years. BDQ is a game-changer for the treatment of rifampicin-resistant tuberculosis (RR-TB) as it enabled the replacement of a long, toxic regimen with a short, all-oral regimen [1–4]. In the context of the COVID-19 pandemic, the importance of the all-oral regimen has been highlighted since it facilitates home-based care for vulnerable TB patients [5]. Currently, the World Health Organization (WHO) recommends BDQ as one of three core drugs that should be included in all RR-TB treatment regimens [4].

Despite the endorsement of BDQ ten years ago, access to BDQ-containing regimens still varies across countries and is impacted by multi-level factors. Structural barriers to BDQ access include the lag in BDQ registration with national regulatory bodies, difficulties in setting up the required pharmacovigilance system, lack of access to companion drugs, and restriction of BDQ to in-patient settings [6–8]. The cost of BDQ also plays a role, especially in high-income countries, with a cost of €23,000 per 6-month course in European countries as compared to €340 in low- and middle-income countries [9, 10]. In addition, the decision to initiate BDQ could also depend on patient characteristics, especially in children, pregnant women or patients with extrapulmonary TB [4], and physician-related factors, particularly their attitude towards innovation, risk and uncertainty [11, 12].

Soon after the introduction of BDQ, resistance was already observed [13, 14]. The emergence of resistance in combination with challenges in assessing resistance to BDQ hinder the effective use of BDQ. Phenotypic drug susceptibility tests (pDST), the current gold standard, take up to six weeks to perform, are not yet standardized and the critical concentration cut-offs are still based on interim recommendations [15–17]. A rapid genotypic DST (gDST) is not yet available for BDQ, in part due to the incomplete understanding of the association between the presence of variants in candidate BDQ resistance-conferring genes and BDQ phenotype. In fact, in the 2021 WHO catalogue of mutations in *Mycobacterium tuberculosis* (Mtb) complex, no variants met the criteria for association with BDQ resistance [18]. To overcome this challenge, a Bayesian approach has been proposed to predict the probability (and 95% credible interval) of a BDQ resistant phenotype from genotypic data [19]. The Bayesian approach is conceptually similar to how clinicians approach the diagnostic process and the selection of a treatment regimen [20, 21].

While promising, it is unclear how physicians would use the probability estimate of BDQ resistance in their decision to prescribe BDQ for the treatment of RR-TB. In this study, we aimed to investigate the decision-making process of physicians in prescribing BDQ in the face of different levels of probability of BDQ resistance. Specifically, we qualitatively explored the perception of physicians on BDQ resistance probability, the factors influencing decision of physicians to prescribe BDQ, and the interaction between BDQ resistance probability and other factors in the decision-making process.

## Methods

### Study design

An exploratory qualitative study was conducted using an interpretative description approach to generate knowledge relevant to the clinical context [22]. This approach is pragmatic, flexible, and allows the combined exploration of lived experience and perception of new knowledge to inform innovation of practice. The conduct and report of this study were guided by the consolidated criteria for reporting qualitative research [23].

### Study participants

Physicians experienced in RR-TB management were purposively selected with maximum variation sampling strategy to obtain broad insight into the phenomenon studied [20]. To include participants as diverse as possible, the selection criteria were related to the burden of multi drug-resistant TB (MDR-TB) (high versus low) and economic status (low, lower-middle, upper-middle or high income) of their country of residence, the cost of BDQ in their country (high versus low) [5, 10], participants’ years of experience in RR-TB treatment (≤ 2 years versus > 2 years), and type of hospital (academic/research hospital versus non-academic hospital). The inclusion of participants was stopped when data saturation was reached.

### Data collection

Prior to the interview, a short, structured questionnaire was sent to the participants to collect information about their demographic characteristics, experience in RR-TB treatment and the use of BDQ in their setting (Additional file 1). Participants also received five hypothetical patient scenarios (Additional file 1) that reflected diverse but realistic patient characteristics, and were asked to decide whether to prescribe BDQ for each patient or not. The scenarios served as the trigger for the interview. Semi-structured online interviews of approximately 1 h were conducted in English, Portuguese, Vietnamese or Amharic by research staff members trained in qualitative research methodology and practice. The questionnaire, patient scenarios and interview guide (Additional file 2) were guided by studies on factors influencing clinical decision making [11, 24–26], WHO guidelines on the treatment of drug-resistant tuberculosis [4], input from an expert panel consisting of members of the Tuberculosis Omics ResearCH consortium [27] and the multidisciplinary research team. During the interview, participants were first asked about their perception and practice of BDQ use. Next, the five patient scenarios were discussed. Participants were asked to explain their clinical decision-making process regarding prescribing BDQ and probed about the influencing factors.

### Data analysis

The interviews were audio recorded, transcribed verbatim, translated to English, and entered into NVIVO Software 1.6.1 for analysis. Thematic analysis was undertaken with a combination of deductive and inductive approaches. A theoretical framework adapted from Research and Development (RAND) Europe was used to guide the deductive coding process [28]. Factors influencing the decision to prescribe BDQ were classified as operating at macro-level (national and international), meso-level (healthcare facilities) and micro-level (patient characteristics and physicians’ perceptions). We also applied an inductive approach to capture unexpected aspects raised by participants and the different ways physicians assign meaning to BDQ resistance probability, a novel concept introduced to the participants by the researchers [29]. Our prior assumption was that BDQ resistance probability will be interpreted as an individual parameter and certain thresholds of high and low probability can be set to guide treatment decisions.

The first set of transcripts was analyzed by two researchers (PHTT and DZA) to develop the codebook, including the name of codes and themes, their definition and examples [30]. Coding was performed after each interview and compared with the code identified from preceding interviews to contrast the views and behaviors across participants and to test emerging explanations within the data [31]. The codes were assigned, articulated, and categorized into themes and subthemes and mapped onto the RAND framework model. The multidisciplinary research team (backgrounds in nursing, pharmacy, molecular and microbiology, epidemiology and social science) held weekly meetings to discuss the emerged themes and adapt the interview guide in order to capture more data on unclear or controversial themes in upcoming interviews. Once consensus was reached on the main content of the codebook, all subsequent transcripts were analyzed by one researcher (PHTT). After the data analysis was finished, two feedback sessions were organized with participants to confirm if the findings of the study resonated with their perspectives and to receive feedback about the findings.

### Trustworthiness

Multiple strategies were employed to assure the trustworthiness of the study. To ensure credibility, patient scenarios were sent to the participants several days before the interview, allowing prolonged participant engagement, familiarization with the new concept of BDQ resistance probability, and sufficient time for reflection on each case [32]. Several participants were known to the researchers in a professional context. The perception of the researcher conducting the interview as a peer with shared knowledge can make participants less reserved and guarded, leading to more genuine data [33]. To maximize conformability, data were discussed among the research team in weekly meetings to avoid investigator bias in analysis and interpretation, and member check was also conducted with participants through two feedback sessions. Transferability was enhanced by purposive sampling of physicians with diverse backgrounds and by describing the characteristics of participants and study settings. Dependability was ensured by detailed documentation of study protocol, data collection and synthesis process, and documentation of the changes and decisions made during the study.

## Results

Between August 2021 and November 2021, 22 physicians were invited, and 14 physicians agreed to participate in the study. Participants had a median age of 48 years, 57% were male, 43% resided in countries with a high burden of MDR-TB, most worked in academic or research environment (78.6%) and had a median of 10 years of experience with RR-TB care (Table 1). Nine of the 14 participating physicians reported that more than 75% of their patients had access to BDQ and four (all from Brazil) reported that BDQ was not yet accessible outside research studies. Participants worked in Belgium (n = 1), the Netherlands (n = 1), the US (n = 1), the UK (n = 1), Brazil (n = 4), South Africa (n = 4), Ethiopia (n = 1) and Vietnam (n = 1). Classification of MDR-TB burden [5], country income level [34], status of BDQ registration [35], national eligibility criteria for BDQ and conditions for BDQ access [36–39], cost of BDQ [10] and access to whole genome sequencing (WGS) for drug resistance profiling [40–43] are listed by country in Table 2.

**Table 1:** Characteristic of 14 study participants

Characteristics		N (%) or median (range)
Age		47.4 (28.0–67.0)
Gender Male		8 (57.1%)
Country classification	High MDR-TB burden, low & lower-middle income country	2 (14.3%)
	High MDR-TB burden, upper-middle income country	4 (28.6%)
	Low MDR-TB burden, upper-middle income country	4 (28.6%)
	Low MDR-TB burden, high income country	4 (28.6%)
Hospital type	Academic/ Research site	11 (78.6%)
	Non-academic/non-research hospital	3 (21.4%)
Years of experience in MDR-TB care		10 (2.0–26.0)
Eligible patients receiving BDQ	0%	4 (28.6%)
	> 75%	2 (14.3%)
	100%	7 (50%)
	Missing	1 (7.1%)

**Table 2:** Characteristics of participant country

	Belgium	The Netherlands	The United States	The United Kingdom	Brazil	South Africa	Vietnam	Ethiopia
Number of participants	1	1	1	1	4	4	1	1
Country income level	High	High	High	High	Upper-middle	Upper-middle	Lower-middle	Low
MDR-TB burden	Low	Low	Low	Low	Low	High	High	High
BDQ registration	Registered	Registered	Registered	Registered	Registered	Registered	Registration pending	Registered
National BDQ eligibility guideline	Not publicly available	Patients with RR-TB	Patients $\ge$ 6 years and weighing $\ge$ 15 kg with pulmonary MDR-TB	Patients $\ge$ 6 years with RR-TB	Patients with RR-TB	Patients $\ge$ 6 years and weighing $\ge$ 15 kg with RR-TB	Patients $\ge$ 6 years with RR-TB and without high risk of cardiovascular complication, severe liver disease and severe electrolyte disorder	Patients $\ge$ 6 years with RR-TB
Conditions for BDQ access	After approval from national TB Consilium	After referral to national reference center	After BDQ order from treating physician is accepted by Metro Medical or Johnson & Johnson Patient Assistance Foundation	After approval from national TB Consilium	As part of operational research (until October 2021)	At all health facilities	Compassionate use after approval from hospital scientific committee	After referral to one of 60 MDR-TB Treatment Initiation Centers
Cost of BDQ (cost for 6 months)	High (~ $22,000)	High (~ $22,000)	High (~ $30,000)	High (~ $22,816)	Low ($340)	Low ($400)	Low ($340)	Low ($340)
Access to WGS for DST	Routine	Routine	Routine	Routine	Research	Research	Research	Research

*BDQ* Bedaquiline, *DST* drug susceptibility testing, *MDR-TB* multi drug-resistant TB, *RR-TB* rifampicin-resistant TB, *WGS* whole genome sequencing

### Structural factors at macro and meso-level influencing BDQ prescription

Physicians stated that their BDQ prescription follows the national guidelines and the WHO guidelines, in which all patients (≥ 6 years) with newly diagnosed RR-TB are eligible to receive BDQ (Table 2). In countries where the limited availability of BDQ was a barrier for eligible patients to access BDQ*,* physicians tended to reserve BDQ for difficult cases.

*Right now I have a little bit of BDQ, but having to choose a patient, I would give it to the patient with a lot of resistance, with a history of treatment abandonment, to try to save this patient. (Physician 06, LB-MI)*

Approval by a clinical advisory committee at the institutional, regional, or national level is a factor controlling the use of BDQ in most countries (Table 2). Discussion with the committee is always required to construct individualized regimens for patients having a complicated treatment history, severe comorbidities, extensively drug-resistant TB (XDR-TB), or extra-pulmonary TB.

*[…]some patients who were already put on treatment and defaulted or lost to follow-up, interrupted the medication and then come back, we always say, ask some advice. The last opinion on comorbidities, I’d say that, someone with, like, renal failure or diabetes mellitus come with RR-TB, I always ask question, ask advice. In case of pre-XDR or XDR patient, with uhm, more with um, extrapulmonary TB, like the TB meningitis with resistance, I always ask for advice of other clinicians. (Physician 09, HB-MI)*

The availability of other RR-TB drugs also influences the prescription of BDQ in the regimen.

*But we have so little alternative that I kept thinking about all these scenarios here. BDQ is entering the market […] alone, so it will be used for all cases indiscriminately. We still have LZD, which can help BDQ, but we needed another drug, DLM, pretomanid... For when you had this kind of scenario, you had a choice, but sometimes we don’t have a choice. (Physician 07, LB-MI)*

The discrepancy in the cost of BDQ between countries influenced the way BDQ was prescribed. In low and lower-middle-income countries where BDQ could be purchased at a reduced price, the cost of BDQ use is not a concern for physicians. In contrast, in high-income countries, BDQ could hardly be withdrawn from the regimen because of its high upfront cost.

*So you can’t, you can’t buy a week or a month when you, when we pay for BDQ, you just get the whole six months. […] I don’t like wasting money. I’m not going to stop this unless I have to, I might add other drugs, I won’t stop it. (Physician 13, LB-HI)*

### Physicians’ perceptions of BDQ features, BDQ resistance and accuracy of BDQ DST

All physicians perceived BDQ as an excellent drug with high efficacy, tolerability, and good treatment outcomes. The value of BDQ especially lies in its enablement of full-oral regimens, shortening treatment duration, and reduction of pill burden, which enhances patient adherence.

*So let’s suppose I had the BDQ here with me right now to get me started. I would initiate everyone I could to replace injectables right away. I wouldn’t think twice and try to make a shorter treatment so that I could reduce the chance of abandonment (Physician 07, LB-MI)*

In general, physicians perceived BDQ as safer than other TB drugs, especially as compared to injectable agents. QT prolongation was the most cited toxicity risk of BDQ, but physicians saw the risk of BDQ as minimal and outweighed by its benefit.

*It's a safe drug. It’s easy to take. It’s not associated with any major adverse events or tolerability issues. So I would tend to use it almost always, even in the face of potential resistance. (Physician 03, HB-MI)*

Four physicians stated that they could comfortably prescribe BDQ in the absence of BDQ DST as they considered the risk of BDQ resistance to be low given its recent introduction in routine care.

*BDQ, it has been researched here, but it’s relatively a new kid on the block with the TB treatment. And a lot of the patients, um, coming from the drug sensitive regimen, haven’t had exposure to it yet, so if we don’t have the drug susceptibility tests towards that, we can just add it, because we know it’s a new drug to the regimen and basically no history of previous exposure. (Physician 02, HB-MI)*

Other physicians took a more prudent view, especially in the presence of poor adherence because of the exceptionally long half-life of BDQ.

*So patients become lost to follow up at an early stage of their treatment, but BDQ remains in the body for a long, long time, if they are still, um, clinically ill and have high number of organisms with only BDQ on board, do you have a very high risk? […] We recently did a study with quite a number of the adults on treatment, uh, became resistant, um, during treatment. And I think one of the reasons for that is, um, adult was not being adherent to treatment and was only BDQ on board. (Physician 05, HB-MI)*

For DST, most physicians did not have an opinion on the accuracy of pDST and gDST for BDQ and simply rely on what the laboratory reports. Some physicians regarded pDST as the gold standard for clinical use, while a physician acknowledged that pDST for BDQ is not yet standardized and may thus not completely accurate. Two physicians expressed skepticism for both gDST and pDST because the laboratory results sometimes contradicted patients’ response to treatment.

*In my opinion, there is still no established standard, right? I’ve already seen the WHO recommendations […] on how to make the MIC and how to interpret it properly, but it’s still a method that still doesn’t have such high quality, but it’s logical that any documented resistance we’ll appreciate. The same thing for molecular biology, there are already several genes that indicate resistance, […] but in practice we are still not sure, it can be like several low- or high-level resistances that do not know if they present in the phenotypic or not. So, I think they're in the process of finding that out, both molecular and phenotypic are not accurate. (Physician 10, LB-MI)*

When pDST and gDST results are discrepant, physicians had varied opinions on what test to rely on. Nine physicians said they solely relied on pDST, one relied more on gDST, and three others followed the DST that reported resistance. Most physicians took other factors such as BDQ exposure history and clinical response into account when judging the DST report.

*The first thing I would do is I would look and see whether BDQ resistance is likely. So, uh, likelihood is increased obvious with previous exposure to BDQ. So if someone’s had interrupted the therapy. Or in the case of exposure, if they’ve been exposed to someone who’s got BDQ resistance. So I would combine what I know of the patient’s history with, uh, the result that I get from the genotype. (Physician 03, HB-MI)*

### Physicians’ perception and interpretation of the BDQ resistance probability estimate

The discordance between phenotypic susceptibility observed in clinical isolates and the resistance probability, along with the wide credible interval, placed some physicians in a quandary.

*Having genetic resistance and not having phenotypic resistance, what does that say to me? Says I can use the drug and it doesn’t seem like such an important thing. So, I saw there that there are confidence intervals that go up to 75%, but a confidence interval varies from a little to a lot. I think what’s missing is really experience.[…] We have never worked with the WGS to make clinical decisions. We are used to making decisions on phenotypic resistance, so I think it is very difficult for the clinician to decide based on that. (Physician 07, LB-MI)*

Three physicians stated that they would only be convinced if the genotypic result always translated into phenotypic resistance (i.e. 100% probability). Because they were used to prescribing BDQ without knowing BDQ DST results, two physicians stated that they may not incorporate the resistance probability into their decision-making and only consider patient-related factors.

*I told you previously that stopping BDQ, according to the WGS, it will not make sense for me. Firstly, I’d say, I will deal with my clinical, my clinical and bacteriological presentation of my patient. (Physician 09, HB-MI)*

One physician would not take resistance probability into account because BDQ is considered as an irreplaceable agent for RR-TB treatment.

*According to what I read in your post, the accuracy rate [of gDST] is only about 70%, not very high. And if the patient is tested for that genotype, it is not necessarily resistant to that drug. I only have BDQ as the latest medicine. So I tend to think that even if the results are resistant, I should still use it. And I monitor the patient’s sputum response after that. (Physician 14, HB-LI)*

There was a wide variation in what physicians considered a low or high resistance probability threshold. Ten physicians would comfortably prescribe BDQ at resistance probabilities of 10–25%. For three physicians who greatly favored BDQ, this threshold could approach 50% or even 70%.

*I like the drug and I think that’s why I’m a bit biased towards it, but I would definitely want this probability to be as high as, as, as maybe 60, 70% before I would say, wow, I think this patient is resistant to BDQ, I won’t use it (Physician 02, HB-MI)*

Most physicians would always exclude BDQ when the resistance probability is around 70%, but this could be as high as 85% or as low as 50% for some physicians. In between the two extremes, physicians referred to a grey zone and regarded BDQ resistance probability as a “dynamic” index or “tossing a coin”. Encountering a BDQ resistance probability in the grey zone prompted physicians to deliberate the risks and benefits of including BDQ given the patient’s characteristics and the number of remaining treatment options.

*I’m not sure. I think it [BDQ resistance probability] is dynamic and would depend again on how, um, how many other options I had compared to, um, how worried I was about side effects. (Physician 11, LB-HI)*

*So how high would it have to be for me to exclude? I think, um, I think something like 30, 40% probably. Maybe a little bit higher because we already know at this point that there’s good clinical improvement. (Physician 11, LB-HI)*

Some physicians advocated for a strategy of keeping BDQ in the regimen without counting on it as a fully active drug and adding other drugs to strengthen the regimen.

*I would still say the BDQ is still very much a core drug and I, and I trust, but if it goes towards 40%, of your, like in your cases, I would say I’m a bit nervous about the drug. I’ll add it, but I don’t know what’s going on. Um, so add other drugs just to support it. (Physician 02, HB-MI)*

Although physicians found it difficult to grasp the concept of BDQ resistance probability, they were open-minded and acknowledged that uncertainty is inevitable in clinical practice.

*In these clinical cases, I made these decisions just because of the probabilities. If I only saw the resistance result of yes or not, I wouldn’t use the BDQ, but as it’s likely, it helped me a lot and I liked that. […] I hope you can actually have such a realistic probability for us to use in practice. We miss it. We make a lot of empirical decisions, and the more real laboratory studies, it helps. (Physician 10, LB-MI)*

To facilitate the interpretation and communication with patients, physicians stated they would need a clear explanation of the scientific basis of the BDQ probability estimate, the meaning of the probability, and its 95% credible interval. Furthermore, they felt that a clinician-friendly-algorithm published as a guideline with level of evidence and recommendation would be helpful.

*You’re going to have to develop a method to make these sequencing results more clinician-friendly, as there are many things to consider. First, for this treatment that is being carried out, do you only have BDQ as the most potent drug? Or so, we don’t know how to use WGS results, and we will need to enter the new generation of sequencing, which maybe is the truth of the 21st century. So, we need to develop a better algorithm so that the clinician can be guided. (Physician 07, LB-MI)*

### Impact of patient characteristics

Treatment response was considered the most influential factor for most physicians, with good clinical and microbiological response being cited by 11 physicians as the main reasons to continue BDQ.

*The main factors that influenced my decision was that the patient clinically was doing well. […]I would definitely say whatever regimen I've got the patient on is actually tolerating and is actually doing well on it. (Physician 02, HB-MI)*

Other physicians found that one month follow-up (i.e. the time when gDST results would become available) is too short to evaluate the microbiological response. Physicians also stressed the importance of investigating alternative reasons for a poor clinical response.

*[…] I would keep BDQ in that situation. A month with a positive 1*+ *bacilloscopy does not mean that it did not work, after all, there is only one month left. There are people who really take longer to get the bacilloscopy negative, and even then, they get cured. (Physician 10, LB-MI)*

Patient exposure to BDQ was a determinant of BDQ treatment decision. Previous treatment with BDQ or having contact with a BDQ-treated patient steered seven physicians away from prescribing BDQ.

*So do I include BDQ here? Um, the sister got BDQ, so at least, you know, there is some rationale, […] maybe it could make sense that there um, was, BDQ resistance. (Physician 11, LB-HI)*

There is variation in the way resistance profile influences the treatment decision. Depending on the drug options left to construct a sufficient regimen, physicians weighed the importance of BDQ resistance probability estimate differently.

*I would also add here that, um, this [the inclusion of BDQ in the regimen] would depend much on the availability of a, um, of other drugs that can still build a very effective regimen, if is a, there’s no chance to salvage the patient without it, maybe even a smaller chance would, you know, justify its use. (Physician 12, LB-HI)*

Several physicians based their decision to prescribe BDQ on the susceptibility of other drugs in the regimen. If the strain has some resistance probability to BDQ but is susceptible to other drugs in the regimen, physicians would often still prescribe BDQ.

*In fact all other drugs in the regimen, they are active, they have no resistance to any other quinolone or injectable, […] as the patient is having a great evolution, there is no resistance at that value here, and in this scenario with five drugs, even if BDQ is partially resistant the other drugs could do the job of helping BDQ. (Physician 07, LB-MI)*

The presence of fluoroquinolone resistance did not play a role in the decision making of two physicians, nine physicians were more likely to prescribe BDQ in case of fluoroquinolone resistance, and one was concerned about the use of BDQ in case of fluoroquinolone resistance as this could increase the risk of BDQ resistance.

*The patient has cavitation, large cavitation, number two, the patient has RIF, INH resistance and he has FQs resistance. My reason [to prescribe BDQ] is the patient already is already pre-XDR patient. (Physician 09, HB-MI)*

*[If WGS shows resistance to FQs] Then I think that the likelihood of BDQ resistance is higher, but I still wouldn’t be reluctant to interrupt it because of the benefits of the drug. (Physician 03, HB-MI)*

Physicians had different views regarding the influence of treatment adherence and social situation on BDQ prescription. For one-third of participants, current or history of non-adherence and social issues such as homelessness and alcohol abuse urged them to not prescribe BDQ due to an increase in the risk of BDQ resistance. In contrast, two physicians preferred BDQ for patients with a history of non-adherence and complex social situations, as an all-oral BDQ-containing regimen would help to improve patient adherence. Other physicians would rather strengthen the regimen by adding other drugs and reinforce patient adherence by hospitalizing the patient or offering support to stabilize the patient’s social situation. During the feedback sessions, physicians agreed that, from an ethical and social viewpoint, adherence should not be a determinant to withhold BDQ.

*So withholding a good drug just because you estimate the patient to be poorly adherent would in my view be very poor medicine. We have to put in the effort to protect your drug, but also to treat the patient. (Physician 01, LB-HI)*

Toxicity of BDQ was not considered a concerning factor. Most physicians would only suspend BDQ in case of QTc ≥ 500 ms or symptomatic QTc prolongation. In cases of less pronounced QTc prolongation, physicians would first stop other QTc-prolonging drugs before stopping BDQ. Similarly, BDQ would be the last drug to be withdrawn from the regimen when the patient has elevated liver enzymes.

*So I wouldn’t be surprised if she had elevated liver enzymes because she’s immunocompromised with tuberculosis. […] Of course I'd look for any other potential causes, but, um, I would only start being anxious about BDQ and at five times upper limit normal. And even then it would be the last thing on my list. (Physician 03, HB-MI)*

A few physicians cited that their treatment decision is influenced by baseline bacillary burden at and absence or presence of cavitary TB. The type of TB only played a role when it involved TB meningitis, as data on central nervous system penetration of BDQ is scarce. Four physicians would keep BDQ as a core drug for TB meningitis treatment while others tended to leave out BDQ, particularly in the context of high BDQ resistance probability.

*If it’s only TB meningitis, then yeah, I would be less likely to use BDQ, I mean, based on my understanding of BDQ penetration into the CNS, I think there’s some data suggesting it’s not great and certainly it’s not labeled that way. So, um, I think taking everything together with this significant chance of resistance [47%], I probably wouldn’t. (Physician 11, LB-HI)*

Pregnancy status, age and body mass index of the patient did not influence the physicians’ decision to use BDQ. HIV status and comorbidities of patient may require physicians to take more precaution with drug-drug interaction, but not necessarily lead to changing the regimen.

*You know, we can actually include BDQ, uh, without causing too much chaos in the ART regimen. I would definitely still add it into the patient, this young child regimen (Physician 02, HB-MI)*

## Discussion

Since 2012, BDQ has become increasingly important for RR-TB treatment [4], but efficient use in routine care is challenging due to the limited availability of standardized BDQ DST worldwide [16, 44]. While DST results are communicated as resistant or susceptible, this is not (yet) possible for BDQ due to lack of confident classification of genotype–phenotype associations [18, 45]. We therefore explored the decision-making process of physicians in prescribing BDQ when data is presented as resistance probabilities accompanied by a 95% credible interval. Physicians saw the clinical value in BDQ resistance probability estimates for patient care but found it challenging to define what they considered as low or high resistance probability. Furthermore, they interpretated and used the probability value in a dynamic rather than a static way, where the interpretation of the resistance probability was influenced by patient characteristics such as response to treatment, drug resistance profile, and BDQ exposure history.

Dealing with uncertainty and probability is an important feature of daily clinical decision-making. The “threshold approach” is often used, in which physicians weigh the risks and benefits of testing and treatment. A disease is ruled out when the probability of disease is lower than the test threshold or treatment is started when the probability is higher than the test-treatment threshold [46]. Our results show that physicians could take a conceptually similar approach regarding the use of BDQ. They would prescribe BDQ when the resistance probability is below a certain threshold or exclude BDQ if the probability is above a certain threshold. Between these two thresholds, physicians would jointly deliberate other patient characteristics to weigh the benefit of continuing BDQ, stopping BDQ, or keeping BDQ but strengthen the regimen with additional drug(s). While the approach of re-enforcing the regimen when BDQ may be compromised was not anticipated by the researchers, this could be an attractive approach. Defining these thresholds for such strategy may however be difficult as there was large variation in the threshold values chosen by participating physicians. Another concern to this strategy is that this may increase the risk of selecting or amplifying BDQ-resistant mutants when BDQ is not sufficiently protected by companion agents [47]. This highlights the need of obtaining comprehensive resistance profiles to guide robust regimen construction, for which next-generation sequencing is a promising solution [48].

At the macro and meso levels, we found that structural factors influenced the interpretation and use of the BDQ resistance probability estimate. The barriers posed by affordability and regulatory issues that have been previously reported [49, 50] were confirmed by some of the participating physicians. This underscores that the programmatic introduction and scale-up of BDQ requires substantial efforts by national and international TB stakeholders to allocate budget,create procurement, monitoring and pharmacovigilance systems for BDQ [51, 52]. The use of committees for approval of BDQ use in individual patients and requirements of referral to specific health care facilities further hindered access to BDQ. Access to companion drugs also influenced the interpretation of the BDQ resistance probability, especially when it narrows down the options to replace or protect BDQ. This confirms the findings of a study on early access to BDQ performed in 2015, where lack of access to companion drugs was the most commonly reported country-level barrier [8]. Guaranteeing adequate procurement of affordable RR-TB drugs is thus essential to translate new policies and guidelines into practice and to accelerate the adoption of new advancements in TB care [53, 54].

At the micro-level, we found that perceptions of physicians and especially patient characteristics influenced the use of BDQ resistance probability estimates. Physicians found it challenging to adopt the BDQ resistance probability into their clinical decision-making because of their limited experience with DST for BDQ, unfamiliarity with gDST and poor understanding of WGS results. The wide confidence interval of the resistance probability currently limits the clinical interpretation of the resistance probabilities value. The increasing knowledge of the molecular basis of resistance in the coming years should improve the clinical usefulness of the BDQ resistance probability value. Once ready to be implemented in routine care, the BDQ resistance probability information should be accompanied by a clear interpretation guide and a clinician-friendly decision support system, such as a computerized treatment recommender recently developed for RR-TB [55]. Regarding patient characteristics, the clinical and microbiological response to treatment, resistance profile and BDQ exposure history emerged as factors that strongly influence the interpretation of BDQ resistance probability value. This is not surprising as these factors have been associated with treatment outcome and risk of the acquisition of BDQ resistance [56]. We found differences in the way patients’ adherence and social situations impact physicians’ treatment decisions. While participants agreed that providing support to reinforce adherence should be the prioritized approach, studies have shown variable effectiveness of these interventions by settings and populations, which could explain the difference in the level of concern of physicians over treatment adherence when prescribing BDQ [57]. Frailty of patients (age, pregnancy, BMI), HIV status, risk of BDQ side-effects and comorbidities, some of which have been associated with treatment outcomes of RR-TB [58], did not influence the decision to prescribe BDQ.

The results of this study should be interpreted in light of its strengths and limitations. Major strengths are the wide range of factors explored, guided by the theoretical conceptual framework of macro-, meso- and micro level factors, and the diversity of participants, including a balance in gender and participants representing high and low burden RR-TB countries as well as low, low-middle, upper-middle- and high-income countries. An important limitation is that participants working in an academic or research setting were overrepresented, which may limit the transferability of findings to non-academic settings. Due to the use of the hypothetical patient scenarios, some patient characteristics may have been discussed more thoroughly than other factors, and some contextual factors may have been omitted. Another limitation is the small sample size. Nevertheless, we only stopped recruitment when sufficient saturation was achieved. Finally, gDST results are required to estimate the probability of resistance to BDQ for individual patients. While next generation sequencing has been approved for surveillance and is used in some high-income countries, this is not yet applied for patient care in high RR-TB burden countries [41, 42, 59, 60]. Furthermore, current culture-based sequencing has a turn-around time that is similar to that of phenotypic DST, limiting the current role of gDST for clinical care. The adoption of the novel BDQ resistance probability approach may only become relevant when rapid molecular tests, such as the targeted sequencing Deeplex assay, become available and will require extensive efforts to build next generation sequencing capacity [61–63].

## Conclusion

The use of BDQ resistance probability is an attractive potential alternative approach for communicating DST results when a binary classification as resistant or susceptible is not (yet) possible. This qualitative study highlights the complex and dynamic features of physicians’ decision-making process in establishing a RR-TB regimen for different levels of BDQ resistance probability. It offers insights into the multi-level factors influencing BDQ prescription for RR-TB. Continued investment in global access to BDQ and companion drugs, development of a rapid molecular test, building next generation sequencing capacity in high burden countries and establishment of a clinical decision support system to guide the decision-making regarding the use of BDQ in the presence of a variant in one of the BDQ-resistance-candidate genes will be essential to achieve the full potential of BDQ for RR-TB treatment.

## Supplementary Information

**Additional file 1.** Questionnaireand patient scenarios

**Additional file 2.** Interviewguide

## Data Availability

The datasets generated and/or analyzed during the current study are available from the corresponding author on reasonable request. The datasets are not publicly available because the transcripts could potentially reveal identifiable information.

## Abbreviations

BDQ: Bedaquiline
FQ: Fluoroquinolone
gDST: Genotypic drug susceptibility test
INH: Isoniazid
MDR-TB: Multi drug-resistant tuberculosis
*Mtb*: *Mycobacterium tuberculosis*
pDST: Phenotypic drug susceptibility test
RAND: Research and development
RR-TB: Rifampicin-resistant tuberculosis
XDR-TB: Extensively drug-resistant tuberculosis
WHO: World Health Organization

## References

[1] Schnippel K, Ndjeka N, Maartens G, Meintjes G, Master I, Ismail N (2018). Effect of bedaquiline on mortality in South African patients with drug-resistant tuberculosis: a retrospective cohort study. Lancet Respir Med.

[2] Borisov SE, Dheda K, Enwerem M, Romero Leyet R, Ambrosio L, Centis R (2017). Effectiveness and safety of bedaquiline-containing regimens in the treatment of MDR- and XDR-TB: a multicentre study. Eur Respir J.

[3] Wang M-G, Wu S-Q, He J-Q (2021). Efficacy of bedaquiline in the treatment of drug-resistant tuberculosis: a systematic review and meta-analysis. BMC Infect Dis.

[4] World Health Organization. WHO consolidated guidelines on tuberculosis. Module 4: treatment - drug-resistant tuberculosis treatment. Geneva 2020. 32603040

[5] World Health Organization. Global tuberculosis report 2020. Geneva; 2020.

[6] Guglielmetti L, Hewison C, Avaliani Z, Hughes J, Kiria N, Lomtadze N (2017). Examples of bedaquiline introduction for the management of multidrug-resistant tuberculosis in five countries. Int J Tuberc Lung Dis.

[7] Gotham D, McKenna L, Frick M, Lessem E (2020). Public investments in the clinical development of bedaquiline. PLoS ONE.

[8] Rodriguez CA, Brooks MB, Guglielmetti L, Hewison C, Jachym MF, Lessem E (2019). Barriers and facilitators to early access of bedaquiline and delamanid for MDR-TB: a mixed-methods study. Public Health Action.

[9] Manalan K, Green N, Arnold A, Cooke GS, Dedicoat M, Lipman M (2020). A cost comparison of amikacin therapy with bedaquiline, for drug-resistant tuberculosis in the UK. J Infect.

[10] Partnership ST. StopTB Partnership’s Global Drug Facility (STBP/GDF) FAQs on bedaquiline price reduction and free goods 2020 [Available from: http://stoptb.org/assets/documents/gdf/drugsupply/2020.07.06%20FAQs%20for%20bedaquiline%20price%20announcement.pdf.

[11] Prosser H, Walley T (2003). New drug uptake: qualitative comparison of high and low prescribing GPs’ attitudes and approach. Fam Pract.

[12] Thomson RG, De Brún A, Flynn D, Ternent L, Price CI, Rodgers H, et al. Health Services and Delivery Research. Factors that influence variation in clinical decision-making about thrombolysis in the treatment of acute ischaemic stroke: results of a discrete choice experiment. Southampton (UK): NIHR Journals Library; 2017. 28151613

[13] Andries K, Villellas C, Coeck N, Thys K, Gevers T, Vranckx L (2014). Acquired resistance of Mycobacterium tuberculosis to bedaquiline. PLoS ONE.

[14] Nimmo C, Millard J, van Dorp L, Brien K, Moodley S, Wolf A (2020). Population-level emergence of bedaquiline and clofazimine resistance-associated variants among patients with drug-resistant tuberculosis in southern Africa: a phenotypic and phylogenetic analysis. The Lancet Microbe.

[15] Kaniga K, Aono A, Borroni E, Cirillo DM, Desmaretz C, Hasan R (2020). Validation of bedaquiline phenotypic drug susceptibility testing methods and breakpoints: a multilaboratory. Multicountry Study J Clin Microbiol.

[16] Köser CU, Maurer FP, Kranzer K (2019). “Those who cannot remember the past are condemned to repeat”: drug-susceptibility testing for bedaquiline and delamanid. Int J Infect Dis.

[17] World Health Organization. Technical Report on critical concentrations for drug susceptibility testing of medicines used in the treatment of drug-resistant tuberculosis. Geneva; 2018. Contract No.: WHO/CDS/TB/2018.5.

[18] World Health Organization. Catalogue of mutations in Mycobacterium tuberculosis complex and their association with drug resistance. Geneva 2021. Available from: https://www.who.int/publications/i/item/9789240028173.

[19] Anlay DZ, Rivière E, Trang Tu PH, Abrams S, Van Rie A. A Bayesian approach to estimate the probability of resistance to bedaquiline in the presence of a genomic variant. bioRxiv. 2022:2022.08.30.505812. 10.1371/journal.pone.0287019 PMC10266631 37315052

[20] Bours MJL (2021). Bayes’ rule in diagnosis. J Clin Epidemiol.

[21] Sox HC, Higgins MC, Owens DK. Understanding New Information: Bayes’ Theorem. Medical Decision Making Second ed: John Wiley & Sons, Ltd.; 2013. p. 61–92.

[22] Thompson Burdine J, Thorne S, Sandhu G (2021). Interpretive description: a flexible qualitative methodology for medical education research. Med Educ.

[23] Tong A, Sainsbury P, Craig J (2007). Consolidated criteria for reporting qualitative research (COREQ): a 32-item checklist for interviews and focus groups. Int J Qual Health Care.

[24] Watt A, Tiessen J, Ling T, Rabinovich L (2007). Prescribing in primary care: understanding what shapes GPs’ prescribing choices and how might these be improved.

[25] van de Berg SEJ, Pelzer PT, van der Land AJ, Abdrakhmanova E, Ozi AM, Arias M (2021). Acceptability, feasibility, and likelihood of stakeholders implementing the novel BPaL regimen to treat extensively drug-resistant tuberculosis patients. BMC Public Health.

[26] Wood F, Simpson S, Butler CC (2007). Socially responsible antibiotic choices in primary care: a qualitative study of GPs’ decisions to prescribe broad-spectrum and fluroquinolone antibiotics. Fam Pract.

[27] TORCH—Tuberculosis Omics ResearCH 2022 [Available from: https://torch-consortium.com/.

[28] Scoggins A, Tiessen J, Ling T, Rabinovich L. Prescribing in primary care, Understanding what shapes GPs’ prescribing choices and how might these be changed. Technical report prepared for the National Audit Office. RAND Corporation …; 2006.

[29] Gale NK, Heath G, Cameron E, Rashid S, Redwood S (2013). Using the framework method for the analysis of qualitative data in multi-disciplinary health research. BMC Med Res Methodol.

[30] DeCuir-Gunby JT, Marshall PL, McCulloch AW (2010). Developing and using a codebook for the analysis of interview data: an example from a professional development research project. Field Methods.

[31] Kahlke RM (2014). Generic qualitative approaches: pitfalls and benefits of methodological mixology. Int J Qual Methods.

[32] Hyde J, Calnan M, Prior L, Lewis G, Kessler D, Sharp D (2005). A qualitative study exploring how GPs decide to prescribe antidepressants. Br J Gen Pract.

[33] Chew-Graham CA, May CR, Perry MS (2002). Qualitative research and the problem of judgement: lessons from interviewing fellow professionals. Fam Pract.

[34] The World Bank. World Bank Country and Lending Groups 2022 [Available from: https://datahelpdesk.worldbank.org/knowledgebase/articles/906519-world-bank-country-and-lending-groups.

[35] Johnson J. Bedaquiline Country Regulatory Status Overview 2021 [Available from: https://www.jnj.com/_document/bedaquiline-country-regulatory-status-overview?id=0000016e-0467-db13-a9ef-566f1a520000.

[36] Nahid P, Mase SR, Migliori GB, Sotgiu G, Bothamley GH, Brozek JL (2019). Treatment of drug-resistant tuberculosis. An Official ATS/CDC/ERS/IDSA Clinical Practice Guideline. Am J Respir Crit Care Med.

[37] NHS England Specialised Services Clinical Reference Group for Infectious Diseases. Treatment for defined patients with MDR-TB and XDR-TB including bedaquiline and delamanid. NHS England; 2021. Contract No.: 201203P.

[38] Jarovsky D (2019). Treatment of tuberculosis in Brazil—past, present, and future challenges. Curr Treat Options Infect Dis.

[39] Ministry of Health—Ethiopia. Guidelines for Clinical and Programmatic Management of TB, TB/HIV, DR-TB and Leprosy in Ethiopia. 7th ed. Addis Ababa, Ethiopia, 2021.

[40] Mugwagwa T, Abubakar I, White PJ (2021). Using molecular testing and whole-genome sequencing for tuberculosis diagnosis in a low-burden setting: a cost-effectiveness analysis using transmission-dynamic modelling. Thorax.

[41] Soetaert K, Ceyssens P-J, Boarbi S, Bogaerts B, Delcourt T, Vanneste K (2019). Retrospective evaluation of routine whole genome sequencing of Mycobacterium tuberculosis at the Belgian National Reference Center. Acta Clin Belg.

[42] Meehan CJ, Goig GA, Kohl TA, Verboven L, Dippenaar A, Ezewudo M (2019). Whole genome sequencing of Mycobacterium tuberculosis: current standards and open issues. Nat Rev Microbiol.

[43] Shea J, Halse TA, Lapierre P, Shudt M, Kohlerschmidt D, Van Roey P (2017). Comprehensive whole-genome sequencing and reporting of drug resistance profiles on clinical cases of Mycobacterium tuberculosis in New York State. J Clin Microbiol.

[44] Farooq HZ, Cirillo DM, Hillemann D, Wyllie D, van der Werf MJ, Ködmön C (2021). Limited capability for testing Mycobacterium tuberculosis for susceptibility to new drugs. Emerg Infect Dis.

[45] Ismail N, Rivière E, Limberis J, Huo S, Metcalfe JZ, Warren RM (2021). Genetic variants and their association with phenotypic resistance to bedaquiline in Mycobacterium tuberculosis: a systematic review and individual isolate data analysis. Lancet Microbe..

[46] Pauker SG, Kassirer JP (1980). The threshold approach to clinical decision making. N Engl J Med.

[47] Nguyen TVA, Anthony RM, Bañuls A-L, Nguyen TVA, Vu DH, Alffenaar J-WC (2017). Bedaquiline resistance: its emergence, mechanism, and prevention. Clin Infect Dis.

[48] Mishra GP, Mulani J (2022). Implications of bedaquiline-resistant tuberculosis. Lancet Infect Dis.

[49] Masini T, Hauser J, Kuwana R, Nhat Linh N, Jaramillo E (2018). Will regulatory issues continue to be a major barrier to access to bedaquiline and delamanid?. Eur Respir J.

[50] Zhang Y, Liu X, Yang L, Zhang G, Gu Z, Chen Z (2020). Barriers and strategies: a review of access to affordable multi-drug resistant tuberculosis medication in China. Infect Drug Resist..

[51] World Health Organization (2015). Introduction of bedaquiline for the treatment of multi-drug resistant tuberculosis at country level: implementation plan.

[52] Furin J, Brigden G, Lessem E, Rich M, Vaughan L, Lynch S. Global progress and challenges in implementing new medications for treating multidrug-resistant tuberculosis. Emerg Infect Dis. 2016;22(3). 10.3201/eid2203.151430 PMC4766879 26885674

[53] Brigden G, Nhung NV, Skrahina A, Ndjeka N, Falzon D, Zignol M (2019). Advances in clinical trial design for development of new TB treatments—translating international tuberculosis treatment guidelines into national strategic plans: experiences from Belarus, South Africa, and Vietnam. PLoS Med.

[54] Tiberi S, Pontali E, Tadolini M, D’Ambrosio L, Migliori GB (2019). Challenging MDR-TB clinical problems—the case for a new Global TB Consilium supporting the compassionate use of new anti-TB drugs. Int J Infect Dis.

[55] Verboven L, Calders T, Callens S, Black J, Maartens G, Dooley KE (2022). A treatment recommender clinical decision support system for personalized medicine: method development and proof-of-concept for drug resistant tuberculosis. BMC Med Inform Decis Mak.

[56] Ismail NA, Omar SV, Moultrie H, Bhyat Z, Conradie F, Enwerem M (2022). Assessment of epidemiological and genetic characteristics and clinical outcomes of resistance to bedaquiline in patients treated for rifampicin-resistant tuberculosis: a cross-sectional and longitudinal study. Lancet Infect Dis.

[57] Pradipta IS, Houtsma D, van Boven JFM, Alffenaar J-WC, Hak E (2020). Interventions to improve medication adherence in tuberculosis patients: a systematic review of randomized controlled studies. Npj Prim Care Respir Med..

[58] Peetluk LS, Ridolfi FM, Rebeiro PF, Liu D, Rolla VC, Sterling TR (2021). Systematic review of prediction models for pulmonary tuberculosis treatment outcomes in adults. BMJ Open.

[59] Lam C, Martinez E, Crighton T, Furlong C, Donnan E, Marais BJ (2021). Value of routine whole genome sequencing for Mycobacterium tuberculosis drug resistance detection. Int J Infect Dis.

[60] World Health Organization. The use of next-generation sequencing technologies for the detection of mutations associated with drug resistance in Mycobacterium tuberculosis complex: technical guide. Geneva. 2018.

[61] Rivière E, Heupink TH, Ismail N, Dippenaar A, Clarke C, Abebe G (2021). Capacity building for whole genome sequencing of Mycobacterium tuberculosis and bioinformatics in high TB burden countries. Brief Bioinform.

[62] Tekola-Ayele F, Rotimi CN (2015). Translational genomics in low- and middle-income countries: opportunities and challenges. Public Health Genomics.

[63] Jouet A, Gaudin C, Badalato N, Allix-Béguec C, Duthoy S, Ferré A, et al. Deep amplicon sequencing for culture-free prediction of susceptibility or resistance to 13 anti-tuberculous drugs. Eur Respir J. 2021;57(3). 10.1183/13993003.02338-2020 PMC8174722 32943401

