# Supplementary material for: Bedaquiline resistance probability to guide treatment decision making for rifampicin-resistant tuberculosis: insights from a qualitative study
Source: BMC Infect Dis. 2022 Nov 22;22:876. doi: 10.1186/s12879-022-07865-7 (PMC9682818; doi:10.1186/s12879-022-07865-7)
Supplement: Supplementary file 1 — Additional file 1. Questionnaireand patient scenarios [file 12879_2022_7865_MOESM1_ESM.pdf]

University of Antwerp

Bedaquiline resistance probability to guide treatment decision making for rifampicin-resistant tuberculosis - insights from a qualitative study

QUESTIONNAIRE AND PATIENT SCENARIOS

I. Background information

Please answer the questions below:

1. Sex:
   ☐ Male
   ☐ Female

2. Age: ___ years

3. Type of hospital you are currently working in:
   ☐ Academic
   ☐ Research site
   ☐ Non-academic/non-research hospital

II. General questions related to the use of bedaquiline (BDQ)

Please answer the questions below:

1. How many years of experience do you have in managing multi-drug resistant tuberculosis (MDR-TB) patients?

   _____ *years*

2. What are the eligibility criteria for BDQ in your setting?

   ____

3. Do all patients in your hospital who meet the eligibility criteria for BDQ receive BDQ?

   ☐ Yes

   ☐ No

   If no: About what proportion of eligible patients gain access to BDQ? ____%

Why do some patients not gain access? ______________________________

4. In the past 6 months, for how many drug-resistant TB patients have you made a treatment decision? ____ patients.

5. Among them, for how many patients have you prescribed BDQ containing regimen? _____ patients.

III. Patient scenarios

In each patient scenario, we present a hypothetical but realistic patient. For each patient, we describe important sociodemographic and clinical characteristics including information related to TB and comorbidities. We present the drug resistance profile based on the whole genome sequencing (WGS) results. For BDQ, the WHO has listed pepQ, Rv0678, mmpL5, mmpS5, atpE, and Rv1979c as candidate genes for BDQ resistance but for none of the variants the information available allowed for a classification as associated with resistance. We used a Bayesian approach to estimate the probability of BDQ resistance. Typically, a Bayesian approach combines different sources of data. We combined the opinions of 33 experts on genotype and phenotype association for BDQ with the data on phenotypic and genotypic BDQ resistance from a recent systematic review. The output of the analysis is the probability of BDQ resistance for a specific genomic variant and the 95% credible interval for that probability. The probability can be interpreted as the prediction that an isolate with that variant will be resistant to BDQ. For example, a 36% probability of BDQ resistance for variant X means that there is a 36% chance that a Mtb isolate containing variant X will be resistant to BDQ and a 64% chance that the Mtb isolate with variant X will be susceptible to BDQ. In addition to the probability estimate, a 95% credibility interval is provided. This credibility interval expresses the uncertainty around the estimate and gives the range in which the truth lies. For example, a 36% probability of resistance with credibility interval 17 to 53% for variant X means that an isolate with variant X is 36% likely to be resistant to BDQ, but there is some uncertainty about this prediction: it may be 17% likely to be resistant or 53% likely to be resistant.

Abbreviations used

AMK: amikacin	MDR-TB: multi-drug resistant tuberculosis
ART: anti-retroviral therapy	MFX: moxifloxacin
BDQ: bedaquiline	*Mtb*: *Mycobacterium tuberculosis*
BMI: body mass index	PZA: pyrazinamide
CFZ: clofazimine	RIF: rifampicin
CXR: chest X-ray	RR-TB: rifampicin-resistant tuberculosis
DLM: delamanid	SLIs: second line injectables
DST: drug susceptibility test	SNP: single nucleotide polymorphism (genomic variant where 1 of the 3 nucleotides in a codon of a gene is different)
DTG: dolutegravir	
EMB: ethambutol	
ETH: ethionamide	TB: tuberculosis
INH: isoniazid	TDF: tenofovir disoproxil fumarate
FQs: fluoroquinolone	3TC: lamivudine
LPA: line probe assay	TRD: terizidone
LVX: levofloxacin	WGS: whole genome sequencing
LZD: linezolid	

PATIENT 1

A 45-year-old woman was diagnosed with drug-susceptible TB in 2018. She was adherent to her first-line treatment and successfully completed her prescribed TB treatment regimen.

In February 2021, she was diagnosed with RR-TB on Xpert. Her BMI was 17 kg/m^2. Her CXR shows infiltration. Sputum smear was positive (1+). She is HIV positive, started ART in 2018 and is now on ART (TDF-3TC-DTG). Her most recent CD4 count result is 45 cells/µl and the viral load is detectable. Blood tests and QT interval are normal. She is not taking any (other) QT prolongation drugs.

She was started on the standard short course BDQ-containing treatment regimen: BDQ + LVX + CFZ + ETH + High dose INH + EMB + PZA and is adherent to the treatment

Her month 1 smear result is negative. Her month 1 culture result is pending. She is responding well to treatment, coughing less and gaining weight. Her blood tests are normal and QT interval is unchanged (430 ms).

Four weeks after the start of treatment, the WGS results are ready. The isolate is

- Resistant to RIF and INH.
- Susceptible to PZA, EMB, FQs, ETH, and SLIs.

Regarding BDQ, a, 337G>A variant was detected in the *Rv0678* gene.

- This SNP is a missense mutation. The majority of experts believe that missense mutations in *Rv0678* frequently confer BDQ resistance.
- Globally, this variant has only been observed in 3 clinical isolates: 1 phenotypically BDQ resistant and 2 phenotypically BDQ sensitive isolates.

Based on this data, the Bayesian analysis predicts a 47% probability of BDQ resistance with a credibility interval of 12% - 84% for an *Mtb* isolate with the 337G>A variant in the *Rv0678* gene.

Do you continue BDQ in the treatment regimen for this patient?

PATIENT 2

A 42-year-old male was diagnosed with RR-TB on Xpert. He has no history of TB treatment.

At diagnosis, his BMI was 19 kg/m^2. CXR showed infiltrations in the right lobe, no large cavities. His smear was positive (1+). He is HIV positive, on ART (TDF-3TC-DTG), recent CD4 count is 370 cells/uL, undetectable viral load. His blood tests and QT interval were normal (430 ms). He is not taking (other) QT-prolongation drugs.

He was started on the standard short-course RR-TB treatment regimen: BDQ + LVX + CFZ + ETH + high dose INH + EMB + PZA.

At the follow-up consultation one month into treatment, he reports that he has been adherent to his TB treatment and is doing well, coughing less. He has gained weight. His blood tests are normal, and QT interval is unchanged (430 ms).

His month 1 smear is negative. The result of the month 1 culture is still pending.

After 5 weeks of treatment, the WGS result is available: the isolate is:

- Resistant to RIF
- Susceptible to INH, PZA, EMB, FQs, ETH and SLIs

Regarding BDQ, a single nucleotide insertion (418_419insG) variant in the *Rv0678* gene is detected:

- This insertion is a frameshift mutation. The majority of experts believe that a frameshift mutation in the *Rv0678* gene frequently or very frequently confers BDQ resistance.
- Specifically, the 418_419insG variant has been observed in 9 clinical isolates globally. The phenotypic BDQ DST of all 9 isolates was susceptible.

Based on this data, the Bayesian analysis predicts a probability of BDQ resistance of 14% with a credibility interval of 1% - 39% for an *Mtb* isolate with a 418_419insG variant in the *Rv0678* gene.

Taking all information for this patient into account, do you continue BDQ as part of the treatment regimen for this patient?

PATIENT 3

A 23-year-old HIV-negative woman was diagnosed with MDR-pulmonary TB in 2019. She was treated with BDQ + MFX + CFZ + ETH + PZA + INH for 2 months, after which she was lost to follow-up.

In June 2021, she was diagnosed with RR-TB on Xpert. Her BMI is 20kg/m^2, her CXR shows small cavities. Sputum smear is positive (2+). Her liver and kidney function are normal. QT interval is also normal (430 ms). She is not taking any (other) QT-prolongation drugs.

She was started on a long regimen containing BDQ + LZD + LVX + TRD + CFZ.

On consultation after 1 month of MDR-TB treatment, the patient says she is adherent to her treatment but is still coughing and has bouts of nausea. She has not gained weight. Her blood tests are normal, and QT interval is unchanged (430 ms).

Her month 1 smear was 1+, and her month 1 culture result is pending.

Two months after the start of treatment, the WGS results are ready. The isolate is:

- Resistant to RIF and INH.
- Susceptible to PZA, EMB, FQs, ETH, and SLIs.
- No variants in genes that may confer resistance to LZD are reported.

Regarding BDQ, a 187A>G variant in the *Rv0678* gene is detected.

- This single nucleotide polymorphism (SNP) is a missense mutation (causing an amino acid change in the encoded protein). The majority of experts believe that missense mutations *in Rv0678* frequently confer BDQ resistance.
- Globally, this variant has only been observed in 2 clinical isolates. The BDQ phenotypic DST of both these isolates was susceptible.

Based on this data, the Bayesian analysis predicts a 35% probability of BDQ resistance with a credibility interval of 4% - 78% for an *Mtb* isolate with a 187A>G variant in the *Rv0678* gene.

Do you continue BDQ in the treatment regimen for this patient?

PATIENT 4

A 70-year-old man was diagnosed with RR-TB by Xpert on 05/02/2021. He has never been diagnosed with TB before. His sister (who lives in the same town) was diagnosed with TB resistant to INH, RIF and AMK in 2017 and was treated with an all-oral short BDQ containing RR-TB regimen. She was not very adherent but did complete her treatment.

At the 70-year-old patient's RR-TB diagnosis, he was HIV negative, BMI was normal (21 kg/m^2) and his CXR showed infiltrations but no cavities. His sputum smear was positive (2+). He is known to suffer from alcohol abuse, but his liver tests are normal.

The man has not yet come back to the clinic to start his treatment and does not answer his phone.

Three weeks after his diagnosis of RR-TB on Xpert, the WGS result is ready. The isolate is

- Resistant to RIF and INH.
- Susceptible to PZA, EMB, FQs, ETH and SLIs.
- No variants in genes that may confer resistance to LZD or DLM are reported.

Because of the exposure to pre-XDR TB, the recommended regimen for this patient is an all-oral long regimen containing BDQ + LZD + LVX + CFZ + TRD.

Regarding BDQ, a 254T>C variant in the *pepQ* gene is detected.

- This SNP is a missense mutation. About half of experts are uncertain whether a mutation in *pep*Q can confer BDQ resistance, 36% believed that a missense mutation in *pepQ* can confer resistance to BDQ and 12% believed that variants in *pep*Q never confer BDQ resistance.
- Globally, the T254C *pep*Q variant has been observed in 2 clinical isolates. Both these samples were susceptible to BDQ on phenotypic DST.

Based on this data, the Bayesian analysis predicts a 25% probability of BDQ resistance with credibility interval of 1% - 67% for an *Mtb* isolate that contains this 254T>C variant in the *pepQ* gene.

One week after the WGS results became available, the man shows up at the clinic and is ready to start his treatment.

Do you start a BDQ-containing regimen for this patient?

PATIENT 5

A 28-year-old man was treated with first-line TB treatment in 09/2020 for pulmonary TB susceptible to RIF and INH. His CXR at that time showed 2 large cavities. He had a BMI of 18 kg/m^2. He has not been very adherent to his treatment. On 01/02/2021, after month 5 of first-line treatment, his smear result was positive (3+) and the LPA showed resistance to RIF and INH. His blood test and QT interval are normal. He is not taking any (other) QT prolongation drugs.

When he comes back to the clinic, the WGS results are also ready. On WGS, the isolate is:

- Resistant to RIF, INH (*katG* mutation), PZA and FQs.
- Susceptible to EMB, ETH and SLIs.
- No variants in genes that may confer resistance to LZD or DLM are reported.

Regarding BDQ, a single nucleotide deletion (193delG) variant in the *Rv0678* gene is detected.

- This variant is a frameshift mutation. The majority of experts believe that a frameshift in *Rv0678* very frequently confers BDQ resistance.
- Globally, this variant has been observed in 5 clinical isolates and in 2 laboratory experiments. The phenotypic DST of all 5 isolates showed susceptibility to BDQ. The 2 laboratory strains were resistant to BDQ.

Based on this data, the Bayesian analysis predicts a 39% probability of BDQ resistance with credibility interval 12% - 71% for an *Mtb* isolate with a 193G deletion in the *Rv0678* gene.

According to the guidelines, the recommended intensive phase regimen for this patient with FQ resistance is BDQ + LZD + DLM + CFZ + TRD + ETH.

Do you start a BDQ-containing regimen for this patient?
