# Supplementary material for: Bedaquiline resistance probability to guide treatment decision making for rifampicin-resistant tuberculosis: insights from a qualitative study
Source: BMC Infect Dis. 2022 Nov 22;22:876. doi: 10.1186/s12879-022-07865-7 (PMC9682818; doi:10.1186/s12879-022-07865-7)
Supplement: Supplementary file 2 — Additional file 2. Interviewguide [file 12879_2022_7865_MOESM2_ESM.pdf]

University of Antwerp

Bedaquiline resistance probability to guide treatment decision making for rifampicin-resistant tuberculosis - insights from a qualitative study

Interview Guide

Time: 00:00

I. Introduction (2 mins)

My name is ____ [name + XX]

Thank you for your time and willingness to participate in the study.

Objective of the study: To explore the clinical decision-making process of physicians in prescribing bedaquiline (BDQ) in the face of different levels of probability of BDQ resistance as determined by whole genome sequencing.

Interview flow:

- Thank you for signing the informed consent and answering the questionnaire.
- The interview consists of 2 parts: (1) general questions then (2) discuss the five patient scenario's

We will do audio and video recordings of this interview for our own analysis and erase the recoding when the study is completed. Okay for you?

TURN ON RECORDER

Time: 00:02

II. General questions (13 mins)

[Participant's personal perception of BDQ in an MDR/RR-TB regimen]

I would like to start with some general questions about your perception and practice in treating MDR/RR-TB patients

1. In the absence of documented BDQ resistance, what do you consider to be the main benefit of including BDQ in an MDR/RR-TB regimen? **(1.5 mins)**

2. In the absence of documented BDQ resistance, what do you consider to be the main risks of including BDQ in an MDR/RR-TB regimen? **(1.5 mins)**

[Local testing practices]

3. In your facility, what drug susceptibility testing (DST) methods are used to determine the resistance profile of patients with TB? **(5 mins)**

3.1. Do you test for BDQ susceptibility on patients with TB?

Probe: what method do you use for BDQ DST?

[Personal opinion of resistance testing for BDQ]

3.2. In your opinion, how accurate is the phenotypic drug resistance testing for BDQ?

3.3. In your opinion, how accurate is the genotypic drug resistance testing for BDQ?

3.4. What would you do if the result of phenotypic and genotypic DST for BDQ discrepant?

3.4.1. What if the genotypic result is R and phenotypic result is S?

3.4.2. What if the genotypic result is S and phenotypic result is R?

[Clinical decision making]

4. What are the main factors influencing your decision to prescribe BDQ for MDR/RR-TB patients? **(2 mins)**
   Probe: Patient characteristics?
   Policy of the facility?
   Availability of BDQ?
   Price of BDQ?
   Guideline?

5. In what case do you want to consult another clinician or a group of clinicians or clinical advisory committee prior to making MDR/RR-TB treatment decisions? **(2 mins)**

   Probe: What characteristics would constitute a 'difficult case'? (If the person mentions this in their answer

Time: 00:15

III. Questions specific to case scenario (30 mins)

I would like to move to the second part of the interview. Recently we have sent you five patient scenarios. Now I would like to discuss your decision around BDQ use in each scenario. Do you have the scenarios with you now? [If yes, continue. If no, the interviewer shares her screen with the scenario (preferred), or you share the scenarios on your screen]

1. PATIENT 1 (7 mins)

A 45-year-old woman was diagnosed with drug-susceptible TB in 2018. She was adherent to her first-line treatment and successfully completed her prescribed TB treatment regimen.

In February 2021, she was diagnosed with RR-TB on Xpert. Her BMI was 17 kg/m^2. Her CXR shows infiltration. Sputum smear was positive (1+). She is HIV positive, started ART in 2018 and is now on ART (TDF-3TC-DTG). Her most recent CD4 count result is 45 cells/µl and the viral load is detectable. Bloods and QT interval are normal. She is not taking any (other) QT prolongation drugs.

She was started on the standard short course BDQ-containing treatment regimen: BDQ + LVX + CFZ + ETH + High dose INH + EMB + PZA and is adherent to the treatment

Her month 1 smear result is negative. Her month 1 culture result is pending. She is responding well to treatment, coughing less and gaining weight. Her blood test is normal and QT interval is unchanged (430 ms).

Four weeks after the start of treatment, the WGS results are ready. The isolate is

- Resistant to RIF and INH.
- Susceptible to PZA, EMB, FQs, ETH, and SLIs.

Regarding BDQ, a, 337G>A variant was detected in the *Rv0678* gene.

- This SNP is a missense mutation. The majority of experts believe that missense mutations in *Rv0678* frequently confer BDQ resistance.
- Globally, this variant has only been observed in 3 clinical isolates: 1 phenotypically BDQ resistant and 2 phenotypically BDQ sensitive isolates.

Based on this data, the Bayesian analysis predicts a 47% probability of BDQ resistance with a credibility interval of 12% - 84% for an *Mtb* isolate with the 337G>A variant in the *Rv0678* gene.

Do you continue BDQ in the treatment regimen for this patient?

Objective

- To capture the influence of advanced-immunosuppression (frail individuals) and drug side effect on prescribing a BDQ-containing regimen.

Factors:

- Adherent to previous and current TB treatment
- Frail (BMI < 17 kg/m2)
- Smear +1
- HIV Positive (uncontrolled viral load, 45 cell/mm3)
- MDR
- On short course BDQ containing regimen
- Well on treatment
- BDQ R probability 47% (12-84%)

Questions:

Do you need one minute to read this patient scenario once again? (If yes, give the participant one minute for each scenario)

3.1. Based on the given information, do you continue BDQ in the treatment regimen for this patient?

For those responding before the interview : *you decided to continue/ stop BDQ containing regimen for this scenario. Is your decision still the same?*

3.2. What are the main factors influencing your decision?

3.3. Would your decision on prescribing BDQ change if:

- This patient was immunocompetent?
- The patient was diagnosed with TB meningitis?
- This patient has elevated liver enzymes 2xULN after one month of treatment?
- This patient has elevated liver enzymes 3xULN after one month of treatment?
- This patient is hospitalized at the time of WGS result available?

- Any other reflections on this scenario?'

Time: 00:22

2. PATIENT 2 (7 mins)

A 42-year-old male was diagnosed with RR-TB on Xpert. He has no history of TB treatment.

At diagnosis, his BMI was 19 kg/m^2. CXR showed infiltrations in the right lobe, no large cavities. His smear was positive (1+). He is HIV positive, on ART (TDF-3TC-DTG), recent CD4 count is 370 cells/uL, undetectable viral load. His blood test and QT interval were normal (430 ms). He is not taking (other) QT-prolongation drugs.

He was started on the standard short-course RR-TB treatment regimen: BDQ + LVX + CFZ + ETH +High dose INH + EMB + PZA.

At the follow-up consultation one month into treatment, he reports that he has been adherent to his TB treatment and is doing well, coughing less. He has gained weight. His blood test is normal, and QT interval is unchanged (430 ms).

His month 1 smear is negative. The result of the month 1 culture is still pending.

After 5 weeks of treatment, the WGS result is available: the isolate is:

- Resistant to RIF
- Susceptible to INH, PZA, EMB, FQs, ETH and SLIs

Regarding BDQ, a single nucleotide insertion (418_419insG) variant in the *Rv0678* gene is detected:

- This insertion is a frameshift mutation. The majority of experts believe that a frameshift mutation in the *Rv0678* gene frequently or very frequently confers BDQ resistance.
- Specifically, the 418_419insG variant has been observed in 9 clinical isolates globally. The phenotypic BDQ DST of all 9 isolates was susceptible.

Based on this data, the Bayesian analysis predicts a probability of BDQ resistance of 14% with a credibility interval of 1% - 39% for an *Mtb* isolate with a 418_419insG variant in the *Rv0678* gene.

Taking all information for this patient into account, do you continue BDQ as part of the treatment regimen for this patient?

Objective

- To capture the influence of low BDQ resistance probability in a clinically well and adherent patient.

Factors:

- Short course BDQ containing regimen
- HIV positive, controlled
- Smear negative
- Rif mono
- Good adherence to current treatment
- Good clinical response to treatment
- 14% (1-39%)

Questions

1.1. Based on the given information, do you continue BDQ in the treatment regimen for this patient?
For those responding before the interview : *you decided to continue/ stop BDQ containing regimen for this scenario. Is your decision still the same?*

1.2. What are the main factors influencing your decision?

1.3. Would your decision on prescribing BDQ change if the patient:

- had been non-compliant during his current TB treatment?
- had MDR-TB instead of RIF mono-resistant TB?
- had abdominal TB?

- had a QT interval of 460 at baseline?
- had a normal QT at baseline but at follow-up had asymptomatic QT prolongation (QT interval of 460 ms) ?
- had a normal QT at baseline but at follow-up had symptomatic QT prolongation with palpitations?
- was a young HIV-positive child (age 8)?

- Any other reflections on this scenario?

Time: 00:29

3. PATIENT 3 (7 mins)

A 23-year-old HIV-negative woman was diagnosed with MDR-pulmonary TB in 2019. She was treated with BDQ + MFX + CFZ + ETH + PZA + INH for 2 months, after which she was lost to follow-up.

In June 2021, she was diagnosed with RR-TB on Xpert. Her BMI is 20kg/m^2, her CXR shows small cavities. Sputum smear is positive (2+). Her liver and kidney function are normal. QT interval is also normal (430 ms). She is not taking any (other) QT-prolongation drugs.

She was started on a long regimen containing BDQ + LZD + LVX + TRD + CFZ.

On consultation after 1 month of MDR-TB treatment, the patient says she is adherent to her treatment but is still coughing and has bouts of nausea. She has not gained weight. Her blood test is normal, and QT interval is unchanged (430 ms).

Her month 1 smear was 1+, and her month 1 culture result is pending.

Two months after the start of treatment, the WGS results are ready. The isolate is:

- Resistant to RIF and INH.
- Susceptible to PZA, EMB, FQs, ETH, and SLIs.
- No variants in genes that may confer resistance to LZD are reported.

Regarding BDQ, a 187A>G variant in the *Rv0678* gene is detected.

- This single nucleotide polymorphism (SNP) is a missense mutation (causing an amino acid change in the encoded protein). The majority of experts believe that missense mutations *in Rv0678* frequently confer BDQ resistance.

• Globally, this variant has only been observed in 2 clinical isolates. The BDQ phenotypic DST of both these isolates was susceptible.<br><br>Based on this data, the Bayesian analysis predicts a 35% probability of BDQ resistance with a credibility interval of 4% - 78% for a *Mtb* isolate with a 187A>G variant in the *Rv0678* gene.<br><br>**Do you continue BDQ in the treatment regimen for this patient?**
**Objective**<br><br>• To capture the influence of previous exposure to BDQ, treatment adherence on prescribing BDQ treatment.
**Factors**<br><br>• Smear +2<br>• Lost on previous BDQ treatment<br>• MDR<br>• Adherent to current treatment<br>• Sign and symptom persist with treatment<br>• Smear +1 on follow up<br>• 35% (4-78%)

Questions

2.1. Based on the given information, do you continue BDQ in the treatment regimen for this patient?

For those responding before the interview : you decided to continue/ stop BDQ containing regimen for this scenario. Is your decision still the same?

2.2. What are the main factors influencing your decision?

2.3. Would your decision on prescribing BDQ change if:

- This patient had successfully completed her previous BDQ-containing regimen?
- The patient hasn't previously been exposed to BDQ-containing regimen?
- The WGS showed resistance to FQ?
- The patient had responded well to the current treatment?
- The smear result was converted at one month?

- The culture result was negative at one month of follow up?

- This patient is 3-months pregnant?
  Probe: In your opinion, how safe is BDQ for pregnant woman?

- Any other reflections on this scenario?

Time: 00:36

4. PATIENT 4 (7 mins)

A 70-year-old man was diagnosed with RR-TB by Xpert on 05/02/2021. He has never been diagnosed with TB before. His sister (who lives in the same town) was diagnosed with TB resistant to INH, RIF and AMK in 2017 and was treated with an all-oral short BDQ containing RR-TB regimen. She was not very adherent but did complete her treatment.

At the 70-year old patient's RR-TB diagnosis, he was HIV negative, BMI was normal (21 kg/m^2) and his CXR showed infiltrations but no cavities. His sputum smear was positive (2+). He is known to suffer from alcohol abuse, but liver tests are normal.

The man has not yet come back to the clinic to start his treatment and does not answer his phone.

Three weeks after his diagnosis of RR-TB on Xpert, the WGS result is ready. The isolate is

- Resistant to RIF and INH.
- Susceptible to PZA, EMB, FQs, ETH and SLIs.
- No variants in genes that may confer resistance to LZD or DLM are reported.

Because of the exposure to pre-XDR TB, the recommended regimen for this patient is an all-oral long regimen containing BDQ + LZD + LVX + CFZ + TRD.

Regarding BDQ, a 254T>C variant in the *pepQ* gene is detected.

- This SNP is a missense mutation. About half of experts are uncertain whether a mutation in *pep*Q can confer BDQ resistance, 36% believed that a missense mutation in *pepQ* can confer resistance to BDQ and 12% believed that variants in *pep*Q never confer BDQ resistance.
- Globally, the 254T>C *pep*Q variant has been observed in 2 clinical isolates. Both these samples were susceptible to BDQ on phenotypic DST.

Based on this data, the Bayesian analysis predicts a 25% probability of BDQ resistance with credibility interval of 1% -67% for a *Mtb* isolate that contains this 254T>C variant in the *pepQ* gene.

One week after the WGS results became available, the man shows up at the clinic and is ready to start his treatment.

Do you start a BDQ-containing regimen for this patient?
**Objective:**<br>• To capture the influence of alcohol abuse, underlying chronic conditions, and contact with patients exposed to BDQ on prescribing a BDQ-containing regimen.
**Factors:**<br>• Contact history with patient treated with BDQ and poorly adherent<br>• Smear +1<br>• XDR fluroquinolone<br>• BDQ R probability 25 % (1-67%)<br>• Alcohol abuse

Questions:

5.1. Based on the given information, do you start a BDQ-containing regimen for this patient?

For those responding before the interview : *you decided to continue/ stop BDQ containing regimen for this scenario. Is your decision still the same?*

5.2. What are the main factors that urge you to that decision?

5.3. Would your decision on prescribing BDQ change if:

- The sister was fully adherent?
- The patient was no contact with BDQ treated patients
- The patient has unstable psychiatric condition?
- The patient has no history of alcohol abuse?
- The patient has painful peripheral neuropathy?

- The patient has diabetes and controlled with oral drugs?

- The patient has poorly controlled diabetes and has foot ulcer complication?

- The patient is severely anemic (HGB = 7g/dl).

- Any other reflections on this scenario?

Time: 00:43

5. PATIENT 5 (7 mins)

A 28-year-old man was treated with first-line TB treatment in 09/2020 for pulmonary TB,susceptible to RIF and INH. His CXR at that time showed 2 large cavities. He had a BMI of 18 kg/m^2. He has not been very adherent to his treatment. On 01/02/2021, after month 5 of first-line treatment, his smear result was positive (3+) and the LPA showed resistance to RIF and INH. His blood test and QT interval are normal. He is not taking any (other) QT prolongation drugs.

When he comes back to the clinic, the WGS results are also ready. On WGS, the isolate is:

- Resistant to RIF, INH (*katG* mutation), PZA and FQs.
- Susceptible to EMB, ETH and SLIs.
- No variants in genes that may confer resistance to LZD or DLM are reported.

Regarding BDQ, a 193delG variant in the *Rv0678* gene is detected.

- This variant is a frameshift mutation. The majority of experts believe that a frameshift in *Rv0678* frequently or very frequently confers BDQ resistance.
- Globally, this variant has been observed in 5 clinical isolates and in 2 laboratory experiments. The phenotypic DST of all 5 isolates showed susceptibility to BDQ. The 2 laboratory strains were resistant to BDQ.

Based on this data, the Bayesian analysis predicts a 39% probability of BDQ resistance with credibility interval 12% - 71% for a *Mtb* isolate with a 193G deletion in the *Rv0678* gene.

According to the guidelines, the recommended intensive phase regimen for this patient with FQ resistance is BDQ + LZD + DLM + CFZ + TRD + ETH.

Do you start a BDQ-containing regimen for this patient?
**Objective**<br>• To capture the influence of bacterial load, number of effective drugs available and vulnerability of patient on prescribing a BDQ containing regimen.
**Factors:**<br>• Non-adherent to first line anti-TB<br>• High bacterial load (smear 3+ and large cavitation)<br>• XDR (fluoroquinolone resistance)<br>• BMI 18 kg/m^2<br>• BDQ Resistance probability 39% (12-71%)

Questions:

4.1. Based on the given information, do you start a BDQ-containing regimen for this patient?

For those responding before the interview : *you decided to continue/ stop BDQ containing regimen for this scenario. Is your decision still the same?*

4.2. What are the main factors that urge you to that decision?

4.3. Would your decision on prescribing BDQ change if:

- The patient had been adherent to his previous TB treatment
- The smear result was negative at baseline?
- The chest X-ray was normal at baseline?
- The infecting strain was FQ susceptible?
- This person is homeless (for Belgian)/ has unstable housing condition (for other countries)?

- This patient has a history of drug abuse?

- This patient is hospitalized at the time of WGS result available?

- Any other reflections on this scenario?

Time: 00:50

IV. Wrap up (5 mins)

1. After going through the scenario and based on your perception, in general, what probability of BDQ resistance would you consider as a high probability? >_____%

2. In general, what probability of BDQ resistance would you consider as a low probability? <_____%

3. Do you have any final remarks or questions for me?

Thank you so much for your time and your informative answers. Your participation has contributed a lot to our understanding of this subject.

[If we have not received their answers for questionnaire]

After the interview, could you send us your answers for the questionnaire that we sent you last week? Thank you very much.

I will end the recording now.

Thank you once again for your collaboration.

ABBREVIATION

BDQ	Bedaquiline
LVX	levofloxacin
CFZ	Clofazimine
ETH	Ethionamide
INH	Isoniazid
EMB	Ethambutol
Z or PZA	Pyrazinamide
RIF	Rifampicin
MOX	Moxifloxacin
LZD	Linezolid
FLQ	fluoroquinolone
MDR/RR-TB	Multi-drug resistant/ rifampicin-resistant tuberculosis
DST	drug susceptibility testing
Xpert	A tool to rapidly diagnose detects *Mycobacterium tuberculosis* complex and resistance to rifampin at the same time
CXR	Chest x-ray
ART	Anti-retroviral therapy
QT interval	a measurement made on an electrocardiogram used to assess some of the electrical properties of the heart
QT prolongation	a disorder of delayed ventricular repolarization, which places patients at risk for ventricular arrhythmias
LPA	Line Probe Assay
WGS	Whole genome sequencing
